# Supplementary material for: Force-Induced Alignment of Nanofibrillated Bacterial Cellulose for the Enhancement of Cellulose Composite Macrofibers
Source: Int J Mol Sci. 2023 Dec 20;25(1):69. doi: 10.3390/ijms25010069 (PMC10778714; doi:10.3390/ijms25010069)
Supplement: Supplementary file 1 [file ijms-25-00069-s001.zip › ijms-2753002-supplementary.pdf]

Supplementary Material

Force-Induced Alignment of Nanofibrillated Bacterial Cellulose for the Enhancement of Cellulose Composite Macrofibers

Ruochun Wang [1], Tetsuo Fujie [2], Hiroyuki Itaya [2], Naoki Wada [2] and Kenji Takahashi [2,*]

[1] Graduate School of Natural Science and Technology, Kanazawa University, Kanazawa 920-1192, Japan

[2] Institute of Science and Engineering, Kanazawa University, Kanazawa 920-1192, Japan

* Correspondence: ktkenji@staff.kanazawa-u.ac.jp

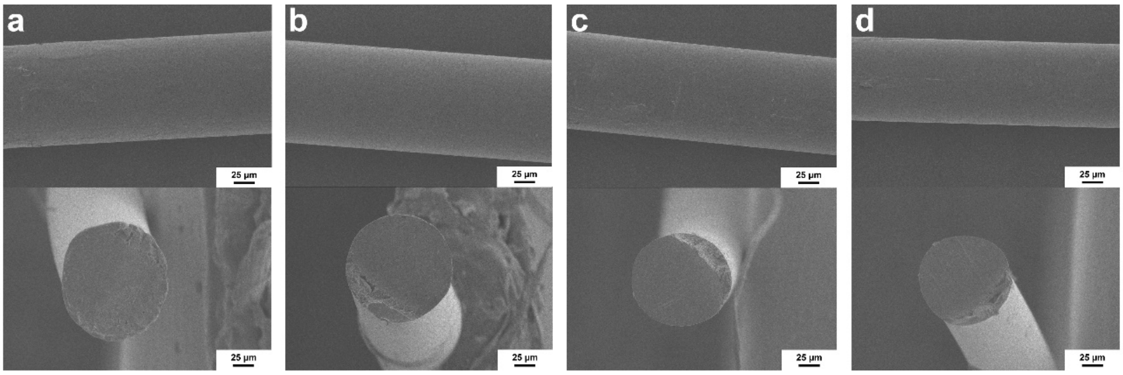

Figure S1. The SEM images of the surface (top) and cross-section (bottom) of HPBC fibers with different stretching ratio: (a) 13%, (b) 26%, (c) 40% and (d) 46%. (Extrusion rate: 30 m min^{-1})

Table S1. The diameters of HPBC fibers with different stretching ratios.

Stretching ratio (%)	0	13	26	40	46
Diameter (μm)	140.7±3.2	138.7±4.2	126.1±1.1	115.7±2.7	108.9±1.9

Table S2. Elemental analysis of HPBC freeze-dried powder and HPBC fiber.

**Sample**	**C (%)**	**H (%)**	**N (%)**
HPBC freeze-dried powder	42.05	6.08	0.26
HPBC fiber	42.05	6.29	0.26

Table S3. The results of tensile strength for HPBC fibers with different extrusion rates and stretching ratios

Strength (MPa)		Stretching ratio (%)				
		0	13	26	40	46
Extrusion rate (m min^{-1})	15	67.2±11.5	69.4±12.0	96.9±22.0	100.0±35.2	110.5±18.3
	20	85.0±12.9	86.5±11.7	107.4±10.1	122.2±27.2	124.7±18.1
	25	96.7±6.9	97.1±8.2	108.8±7.4	125.2±16.6	149.2±26.6
	30	91.5±17.1	88.2±29.8	119.0±19.2	142.6±9.0	173.8±8.3

Table S4. The results of Young's modulus for HPBC fibers with different extrusion rates and stretching ratios.

Young's modulus (GPa)		Stretching ratio (%)				
		0	13	26	40	46
Extrusion rate (m min^{-1})	15	5.3±0.3	6.5±0.7	8.1±1.1	8.8±1.1	8.4±0.9
	20	7.7±0.8	7.2±0.5	8.9±0.5	10.3±0.7	9.7±0.8
	25	7.3±0.7	7.2±0.4	8.3±0.7	9.5±1.1	11.1±0.6
	30	7.7±0.6	7.5±2.9	9.3±0.9	12.0±0.4	13.7±1.3
